# Brucella Peritonitis in a Patient on Peritoneal Dialysis: A Case Report and Review of Literature

**DOI:** 10.7759/cureus.20679

**Published:** 2021-12-25

**Authors:** Saeed M Al Zabali, Aljawharah K Rubaihan, Madawi F Alnetaifat, Salem Alshahrani, Moza Alhammadi

**Affiliations:** 1 Pediatric Nephrology, King Fahad Medical City, Riyadh, SAU; 2 College of Medicine, Al-Maarefa University, Riyadh, SAU; 3 College of Medicine, King Saud Bin Abdulaziz University for Health Sciences, Riyadh, SAU; 4 College of Medicine, King Khalid University, Asir-Abha, SAU; 5 Pediatric Infectious Diseases, Dubai Hospital, Dubai Health Authority, Dubai, ARE; 6 Pediatric Infectious Diseases, King Fahad Medical City, Riyadh, SAU

**Keywords:** end-stage renal disease, catheter removal, peritoneal dialysis, peritonitis, brucella

## Abstract

Peritoneal dialysis (PD)-associated peritonitis is the most common cause of morbidity, mortality, and treatment failure in patients on PD. Brucellosis is a worldwide zoonotic infectious disease caused by gram-negative bacteria of the genus *Brucella*. It is a major public issue in some regions. According to the World Health Organization report in 2011, the Kingdom of Saudi Arabia is considered endemic for brucellosis. *Brucella* peritonitis is one of the rarest presentations of *Brucella*. We report a case of a 14-year-old girl known to have end-stage renal disease, secondary to the autosomal recessive polycystic kidney. She had congenital hepatic fibrosis and pancytopenia. She had been undergoing automated PD for the past seven years and presented with abdominal pain, seizure, and poor feeding. There was no history of ingestion of unpasteurized milk or contact with raw infected animal products. The color of PD fluid was turbid with leukocytosis, predominantly neutrophils. The peritoneal fluid culture was positive for methicillin-resistant *Staphylococcus aureus*. The patient was started on intraperitoneal vancomycin, which showed slow improvement. The second culture of the peritoneal fluid showed *Brucella* species after a few days. Blood culture and serum serology titer for *Brucella* showed negative results. An anti-*Brucella* regimen, including rifampin and doxycycline, was initiated. She was treated with this regimen for six weeks. After the initiation of the anti-*Brucella* regimen, she showed marked improvement. To the best of our knowledge, only a small number of cases of *Brucella* peritonitis in PD patients have been reported. Despite the rarity of *Brucella* as a peritonitis-causing organism, it should be considered as a relevant pathogen in peritonitis cases, especially in endemic regions. PD-associated *Brucella *peritonitis is rare, and PD catheter saving may be considered if there is a response to anti-*Brucella* treatment.

## Introduction

Peritoneal dialysis (PD) is the primary modality of renal replacement therapy in children [1]. The number of patients with end-stage renal disease (ESRD) receiving PD therapy has been increasing because of the improvement in PD techniques and patient survival [2]. PD-associated peritonitis is a major cause of hospitalization in pediatric patients [3]. Peritonitis is the most common complication of PD; it is generally caused by coagulase-negative *Staphylococcus* and *Staphylococcus aureus* [4]. Brucellosis, also known as “undulant fever” or “Mediterranean fever,” is a zoonotic infectious disease transmitted primarily by direct or indirect contact with infected animals or their products. Infection generally occurs in endemic areas [5]. Brucellosis is endemic in Saudi Arabia, with an incidence of 12.44 cases per 100,000 population as reported in 2019 [4]. The bacterium *Brucella* first infects animals and is then transmitted to humans through contact with an infected animal [6]. Brucellosis generally presents as an acute or subacute infection. Hepatosplenomegaly, fever, and peripheral arthritis are the most frequent clinical findings [5]. Peritonitis caused by brucellosis is considered to be rare. Most reported cases were in Saudi Arabia and Turkey, with only one case reported in China. Herein, we present a case of *Brucella*-related PD in a patient who was treated with doxycycline and rifampin for six weeks without the removal of the PD catheter.

## Case presentation

The patient is a 14-year-old Saudi girl with congenital hepatic fibrosis with pancytopenia and ESRD, secondary to autosomal recessive polycystic kidney disease, on automated PD for seven years. She had a medical history of seizure disorders and developmental delay and was on antiepileptic drugs. She was presented to the emergency department with complaints of abdominal pain for one day and a change in the peritoneal dialysate color, associated with abnormal movements described as generalized tonic-clonic convulsions. She had no fever or vomiting, but there was a history of acquiring bacterial peritonitis infections several times. She received a blood transfusion a few months before the presentation and was not known to have any allergies. Her vital signs at admission were as follows: temperature: 37.2°C, heart rate: 106/min, respiratory rate: 24/min, and blood pressure: 97/32 mmHg. On examination, she was conscious and alert but irritable and appeared pale. Her abdomen was distended with tenderness throughout. Other systemic examinations were unremarkable. Upon presentation, the patient’s white blood cell (WBC) count was low at 3.09 10e9/L, along with a high neutrophil proportion of 79.6%, low lymphocyte proportion of 11.0%, a low hemoglobin level of 8.7 g/dl, low platelet count of 61.0 10e9/L (it is the baseline for this case), markedly elevated plasma creatinine level of 675 mmol/L, high urea level of 21 mmol/L, bicarbonate (CO2) level of 18 mmol/L, elevated C-reactive protein level of 157 ml/L, high alkaline phosphatase level of 909 U/I, high alanine aminotransferase level of 98 U/I, and a high bilirubin level of 16.4 umol/L.

A sample of peritoneal fluid was collected for analysis, which exhibited a turbid yellowish appearance, a WBC count of 59,658/mm^3^, and a high neutrophil proportion of 94%. The peritoneal fluid culture was positive for methicillin-resistant *S. aureus*. The patient was started on intraperitoneal vancomycin and ceftazidime, but the abdominal pain did not decrease with the treatment, and she had one spike of fever of 38.1°C. Fungal culture was negative and the second culture of peritoneal fluid revealed *Brucella* species after four days of growth (Table 1).

**Table 1:** Results of peritoneal fluid culture.

Culture	Organism
*Brucella* spp.	
Antibiotic sensitivity	Interpretation result
Trimethoprim/sulfamethoxazole	Sensitive
Gentamicin	Sensitive
Streptomycin	Sensitive
Tetracycline	Sensitive
Doxycycline	Sensitive

Blood culture and serum serology (enzyme-linked immunosorbent assay [ELISA] assay test) for *Brucella* showed negative results. When we further examined the patient’s history, the mother denied any history of ingestion of unpasteurized milk or contact with raw infected animal products. The infectious diseases team was consulted and they started a treatment regimen for brucellosis with oral rifampicin 10 mg/kg once daily and doxycycline 2 mg/kg/dose every 12 hours for six weeks. Blood culture showed negative results for *Brucella*, and a repeat peritoneal culture showed negative findings on the fourth day after treatment initiation. Peritoneal fluid analysis showed a decreased WBC count of 40,000/mm^3^ and clear peritoneal fluid. Although removal of the PD catheter was indicated, the patient’s mother refused as the patient showed clinical improvement. She was discharged in a good condition without the removal of the PD catheter, and PD was resumed as usual without stopping it.

## Discussion

*Brucella* species are gram-negative unencapsulated, nonmotile, nonspore-forming, facultative intracellular bacilli [7]. They are transmitted to humans from infected animals through ingestion, inhalation, conjunctiva, or skin abrasions. This bacterium affects multiple organs in the body through hematogenous spread [8]. The accurate diagnosis of brucellosis is difficult because of nonspecific clinical characteristics, slow growth in cultures, and the complexity of serological diagnosis [9]. Bone and joint involvement is the most frequent complication of brucellosis. According to the International Society for Peritoneal Dialysis, peritonitis must always be diagnosed when at least two of the following findings are present: (a) clinical features consistent with peritonitis, (b) dialysis effluent WBC count of >100/μL or >0.1 × 109/L with >50% polymorphonuclear cells, and (c) positive dialysis fluid culture [10].

Patients who are on PD are at a higher risk of developing peritonitis than the general population because of the impaired peritoneal defenses in patients on PD [11]. *Brucella* shunt infection complicated by peritonitis has been reported [12]. PD-associated *Brucella* peritonitis is extremely rare [8]. To our knowledge, only nine cases have been reported in the literature to date. The majority of the reported cases were in Turkey and Saudi Arabia, with one case reported in China. Table 2 summarizes the general characteristics and clinical findings of the reported cases of *Brucella* PD-related peritonitis. Almost all the reported cases were adult patients (Table 2). Our patient is the only pediatric case.

**Table 2:** Reported cases of Brucella PD-related peritonitis. PD: peritoneal dialysis; p.o.: per oral; IP: intraperitoneal.

Study		Taskapan et al. (2002) [7]	Ozisik et al. (2006) [11]	Alothman et al. (2008) [13]	Unal et al. (2009) case 1 [8]	Unal et al. (2009) case 2 [8]	Solak et al. (2012) [14]	Koz et al. (2014) [15]	Niu et al. (2018) [16]	Bukhari et al. (2018) [17]	Present report
Age, gender		47-year-old male	39-year-old woman	67-year-old male	38-year-old male	52-year-old male	48-year-old man	49-year-old man	54-year-old woman	45-year-old man	14-year-old woman
Country		Turkey	Turkey	Saudi Arabia	Turkey	Turkey	Turkey	Turkey	China	Saudi Arabia	Saudi Arabia
History		15-day history of fatigue, fever, sweating, back pain, and two-day history of cloudy dialysate	Nausea and severe abdominal pain. History of four peritonitis episodes	Change of peritoneal fluid color, associated with abdominal pain and increased lower limb edema of one week	Two-day history of nausea, vomiting, abdominal pain, and cloudy dialysate	History of nausea, vomiting, fever, joint pain, severe abdominal pain, and cloudy dialysate	Abdominal bloating and constipation of two weeks, no fever or hypotension, mild abdominal tenderness	Abdominal pain and cloudy dialysate effluent. He had myalgia and malaise for 10 days	Abdominal pain, nausea, vomiting, diarrhea, fatigue, anorexia, bilateral knee pain, cloudy PD effluent, and ultrafiltration decrease, only one previous peritonitis episode	Two to three days history of fever, on-off vague generalized dull abdominal pain, vomiting, and diarrhea	Abdominal pain for one day and history of change peritoneal dialysate color, no history of fever
Examination		Temperature of 38.2°C, heart rate of 106 beats/min, and blood pressure of 150/100 mmHg, with prominent abdominal tenderness	Temperature of 37.3°C, blood pressure of 160/90 mmHg, and pulse rate of 105 beats/min. The patient had abdominal pain during palpation, and the peritoneal catheter was normal	Abdominal examination revealed diffuse mild tenderness with clean CAPD catheter exit-site. There was bilateral lower limb edema	Temperature of 37.3°C, heart rate of 88 beats/min, blood pressure of 135/85 mmHg, and diffuse abdominal mild tenderness	Temperature of 37.2°C, heart rate of 94 beats/min, blood pressure of 150/100 mmHg. The patient was pale with diffuse abdominal mild tenderness without rebound tenderness or guarding. He also had bilateral orbital edema	No fever or hypotension, mild abdominal tenderness. Other aspects of the physical examination were unremarkable. Peritoneal effluent was not cloudy	No fever. Physical examination revealed abdominal tenderness, negative rebound tenderness	Temperature of 36.2°C, blood pressure of 150/80 mmHg. The patient had umbilical tenderness during palpation and slight bilateral lower limb edema	Temperature was 38°C, blood pressure was 120/60 mmHg, and tender abdomen with turbid PD fluid	Vitally she was hypotensive afebrile. On examination she was irritable, the abdomen was distended with tenderness all over, other systemic examinations were unremarkable
Risk factors		History of unpasteurized, unsalted cheese ingestion	Direct contact with animal	History of raw milk ingestion	History of unpasteurized cheese ingestion	History of unpasteurized milk and cheese ingestion	Contact with sheep and cattle	Not reported	Possible ingestion of undercooked beef, single PD exchange while in restaurant	History of unpasteurized cheese ingestion	No history of direct contact with animal or raw milk ingestion
Time to peritonitis		12 months	5 years	4 months	2 months	6 months	3 years	Not reported	11 years	3 years	7 years
Blood test	Culture	Brucella melitensis on the sixth day	No growth	No growth	Brucella melitensis on the fifth day	Brucella organism on the sixth day	No growth	Not reported	No growth	Isolated heavy growth of Brucella melitensis on the fourth day	No growth
	WBC	6200/ mm3	14.5	8.1 × 109 cells/L	4080/mm3	8100/mm3	7.16 × 109 cells/L	7100/ mm3	17.16 × 109 cells/L	15.9 × 109 cells/L	3.09 10e9/L
PD fluid analysis	Culture	Brucella melitensis on the sixth day	Brucella melitensis	Grew Brucella spp. on day six	Brucella melitensis on the fifth day	Brucella organism on the sixth day	Brucella spp.	Brucella spp.	Brucella spp.	Isolated heavy growth of Brucella melitensis on the fourth day	Brucella spp.
	WBC differential	NA	Neutrophils predominance	8% lymphocytes and 85% neutrophils	Neutrophil predominance	Neutrophil predominance	Lymphocytes	28.9% neutrophils and 70.4% lymphocytes.	Neutrophils 87%	Neutrophils 86%	Neutrophils 94%
	WBC count	300/mm3	3140	3356	1600/mm3	5100/mm3	820	1300/mm3	1950/μL	2280 × 109 cells/L,	59658/cumm
Brucella agglutination test		1/2560	Serum 1/160, dialysate 1/60	Serum 1/2560, dialysate unknown	Serum 1/640, dialysate negative	1/640	1/5120	1/1280	Not reported	Unknown	Negative
Treatment		Doxycycline p.o. and rifampicin p.o. for six weeks	Rifampin p.o. and doxycycline p.o. for six weeks	Doxycycline p.o. and rifampicin p.o. for 12 weeks	Doxycycline p.o. and rifampicin p.o. for six weeks	Doxycycline p.o. and rifampicin p.o. for six weeks	Rifampicin p.o. ceftriaxone for 45 days, doxycycline was not tolerated	Amikacin intraperitoneal (IP), doxycycline p.o., and rifampicin p.o. for 42 days	Levofloxacin IP and amikacin IP for three weeks then minocycline p.o., rifampicin p.o., and levofloxacin p.o. for a total treatment duration of 18 weeks	Rifampicin p.o., doxycycline p.o., ciprofloxacin p.o. Rifampicin was discontinued and doxycycline was replaced by minocycline for 12 weeks	Oral rifampicin and oral doxycycline
Outcome		PD catheter removed. The patient was switched to hemodialysis	PD catheter removed. The patient was switched to hemodialysis	PD catheter removed. The patient was switched to hemodialysis	PD was resumed as usual	PD was resumed as usual	PD was resumed as usual	PD was resumed as usual	PD was resumed as usual	PD catheter removed. The patient was switched to hemodialysis	PD was resumed as usual

Regarding clinical presentations, the most common presenting symptom was abdominal pain. Fever was present in four cases. Our patient presented both fever and abdominal pain. Most cases reported that there was a source for infection with *Brucella*, whereas in our case, the source of infection was not clear, with no obvious environmental risk factors. Her mother denied any history of ingestion of unpasteurized milk or contact with raw infected animal products, but she had received a blood transfusion a few months before presentation. The risk of transfusion-transmitted brucellosis has been reported [18]. Serology is the preferred method for diagnosing brucellosis when bacterial isolation is not possible, and serological testing is widely used in the diagnosis of brucellosis [19]. Turan Buzgan et al. reported that 1.1% of the patients remained seronegative [19]. As the peritoneal fluid showed a positive result for *Brucella* species after four days of growth, and the serum serology titer for *Brucella* revealed negative findings, we diagnosed the patient with seronegative *Brucella* peritonitis.

The PD catheter was removed in four patients with *Brucella* peritonitis [6,11,17]. However, our patient was successfully treated with antibiotics without the removal of the PD catheter. Niu et al. suggested that catheter removal should be considered for those patients with severe manifestations, who are unresponsive despite optimal treatment with intraperitoneal antibiotics and appropriate oral antibiotics [16]. Several studies have shown that it is necessary to use antibiotics for at least six weeks or more to avoid relapse or develop resistance [6]. The best results are observed when antimicrobial treatment is administered early in combination therapy with adequate dosing [16]. The treatment commonly used for children is age-specific. If the patient is aged greater than eight years, the treatment is complex, consisting of oral doxycycline and rifampicin. Children aged less than eight years are routinely treated with trimethoprim, sulfamethoxazole, and rifampicin [6,20]. In most of the reported cases, doxycycline and rifampicin were administered. Levofloxacin or ciprofloxacin was added in some cases [16,17]. Ceftriaxone was administered to one patient, who could not tolerate doxycycline [14]. In our patient, oral rifampicin and doxycycline were highly effective.

## Conclusions

In conclusion, *Brucella* peritonitis in a PD patient is rare but associated with excellent outcomes if treated appropriately. In PD-related peritonitis that does not respond to standard antibiotic treatment, *Brucella* peritonitis should be considered, particularly in endemic countries. The use of oral antibiotics is a good option for treatment unless the patient cannot tolerate it.
